# Longitudinal associations between use of antihypertensive, antidiabetic, and lipid-lowering medications and biological aging

**DOI:** 10.1007/s11357-023-00784-8

**Published:** 2023-04-10

**Authors:** Bowen Tang, Xia Li, Yunzhang Wang, Arvid Sjölander, Kristina Johnell, Madhav Thambisetty, Luigi Ferrucci, Chandra A. Reynolds, Deborah Finkel, Juulia Jylhävä, Nancy L. Pedersen, Sara Hägg

**Affiliations:** 1 Department of Medical Epidemiology and Biostatistics, Karolinska Institutet, 171 77 Stockholm, Sweden; 2 School of Public Health and Emergency Management, Southern University of Science and Technology, Shenzhen, China; 3 Department of Clinical Sciences, Danderyd Hospital, Karolinska Institutet, Solna, Sweden; 4 Brain Aging and Behavior Section, National Institute on Aging, Baltimore, USA; 5 Longitudinal Studies Section, National Institute on Aging, Baltimore, USA; 6 Department of Psychology, University of California, Riverside, CA USA; 7 Aging Research Network-Jönköping (ARN-J), School of Health and Welfare, Jönköping University, Jönköping, Sweden; 8 Center for Economic and Social Research, University of Southern California, Los Angeles, CA USA; 9 Faculty of Social Sciences (Health Sciences) and Gerontology Research Center (GEREC), University of Tampere, Tampere, Finland

**Keywords:** Antihypertensive, Antidiabetic, Lipid-lowering medications, Biomarkers of biological aging

## Abstract

Aging is a major risk factor for many chronic diseases. This study aimed to examine the effects of antihypertensive, lipid-lowering, and antidiabetic drugs on biological aging. We included 672 participants and 2746 repeated measurements from the Swedish Adoption/Twin Study of Aging. Self-reported medicine uses were categorized into antidiabetic, antihypertensive, and lipid-lowering drugs. A total of 12 biomarkers for biological aging (BA biomarkers) were included as outcomes. Conditional generalized estimating equations were applied conditioning on individuals to estimate the drug effect on BA biomarker level within the same person when using or not using the drug. Chronological age, body mass index, smoking status, number of multiple medication uses, blood pressure, blood glucose level, and apoB/apoA ratio were adjusted for as covariates in the model. Overall, using antihypertensive drugs was associated with a decrease in one DNA-methylation age (PCGrimAge: beta = − 0.39, 95%CI = − 0.67 to − 0.12). When looking into drug subcategories, calcium channel blockers (CCBs) were associated with a decrease in several DNA-methylation ages (PCHorvathAge beta = − 1.28, 95%CI = − 2.34 to − 0.21; PCSkin&bloodAge beta = − 1.34, 95%CI = − 2.61 to − 0.07; PCPhenoAge beta = − 1.74, 95%CI = − 2.58 to − 0.89; PCGrimAge beta = − 0.57, 95%CI = − 0.96 to − 0.17) and in functional biological ages (functional age index beta = − 2.18, 95%CI = − 3.65 to − 0.71; frailty index beta = − 1.31, 95%CI = − 2.43 to − 0.18). However, the results within other drug subcategories were inconsistent. Calcium channel blockers may decrease biological aging captured by the BA biomarkers measured at epigenetic and functional level. Future studies are warranted to confirm these effects and understand the underlying biological mechanisms.

**Supplementary Information:**

The online version contains supplementary material available at 10.1007/s11357-023-00784-8.

## Introduction

Aging is a strong risk factor for many chronic disorders [1]. With rapidly aging populations worldwide, particular attention has been paid to the concept of geroprotection, which is an intervention targeting biological aging processes to prevent multiple age-related diseases [2]. Today, more than 400 chemicals have been reported to slow down aging or increase lifespan in various laboratory model organisms [3, 4], but there are limited data available for humans. One major reason is that studies examining human longevity are extremely lengthy and costly. An alternative approach is to use a surrogate endpoint, such as biomarkers that are capable of predicting remaining lifespan and tracking changes in the biological aging process, which are accordingly termed biomarkers of biological aging (BA biomarkers) [5].

In the recognition of the complex aging mechanisms [1], a variety of BA biomarkers have been developed, including telomere length, algorithms applied to genome-wide DNA methylation data, and composites of multiple physiological or functional measures [6]. So far, these BA biomarkers have been examined extensively and have shown good, but heterogeneous, performance in predicting mortality and age-related disorders including cancers, cardiovascular diseases, type 2 diabetes, and Alzheimer’s disease [5–7]. In contrast to chronological age (CA) that increases at the same rate and is irreversible, BA biomarkers have been found to be modifiable in intervention studies [8–11]. In particular, these studies found that reduction in BA biomarkers was accompanied by decreased number of senescent immune cells [9], protective immunological changes, and improved risk indices for many age-related diseases [10]. This synchronization between changes in BA biomarkers and hallmarks of aging supports the validity of BA biomarkers as predictors of biological aging process and their practical use in the discovery of geroprotectors.

Antihypertensive, antidiabetic, and lipid-lowering drugs are three of the most commonly used drugs among middle-aged and older individuals [12]. In addition to their primary roles in regulating blood pressure and metabolism of glucose and lipids, the use of these medications has also been shown to reduce the risk for age-related diseases such as dementia and cancers [13–15]. Particularly, some of these medications have been reported to extend the lifespan of laboratory model organisms [16–20], whereas their effects on human aging are still understudied. So far, only two studies have investigated the relationship between medications and DNA-methylation ages (DNAmAges) [21, 22]. However, both studies relied on longitudinal data collected in two measurements with a mean follow-up duration of five years, which might not be sufficient to capture the change of DNAmAges caused by medication use. In addition, BA biomarkers constructed by various approaches may capture different aspects of the aging process [7, 23]. DNAmAges by itself may not provide a complete picture of the impact of these drugs on aging, making it necessary to consider multiple BA biomarkers that have been measured using different approaches.

Therefore, this study aimed at investigating the associations between antihypertensive, antidiabetic, and lipid-lowering medication use and 12 BA biomarkers. These BA biomarkers were measured longitudinally from molecular to functional levels in the Swedish Adoption/Twin Study of Aging (SATSA) across 20 years of follow-up. We applied a statistical method conditioning on individuals to estimate the drug effect on BA biomarker level within the same person when using or not using the drug. The approach is similar to a case-crossover or self-controlled design, thereby avoiding comparison between different groups of individuals and controlling for individual-constant confounders [24].

## Methods

### Study population

**Fig. 1:**
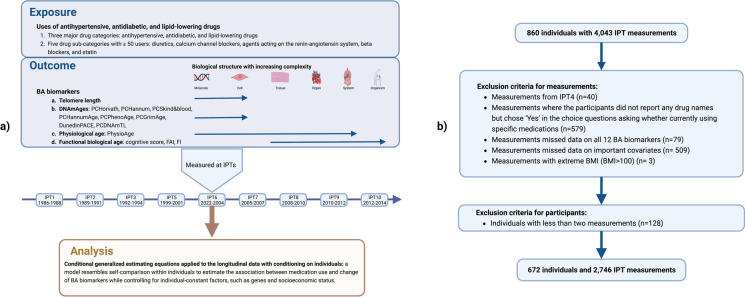
Study design and exclusion criteria for the present study. **a** A conceptual plot for the study design. The arrows in the outcome panel indicates the biological structure levels at which different BA biomarkers were measured. **b** Exclusion criteria for the present study. BA biomarkers indicates biomarkers of biological aging; FAI, functional age index; FI, frailty index; IPT, in-person testing; BMI, body mass index

SATSA is a longitudinal cohort consisting of pairs of twins in Sweden that have been reared together or apart [25]. The participants aged 50 years old or above in SATSA were invited to an in-person testing (IPT) administrated approximately every 3 years. A questionnaire interview, a health examination (fasting blood and morning urine samples were collected), and tests on functional and cognitive abilities were performed in each IPT, except for IPT4 which was performed through telephone interview. A total of 10 IPTs were completed from 1986 to 2014, which generated 4,043 IPT measurements from 860 participants. After exclusions, 672 individuals with 2746 measurements were included in the analysis. See study design and exclusion criteria in Fig. 1.

### Assessment of biological age biomarkers

At each IPT in SATSA, multiple BA biomarkers were measured to quantify different aspects of biological aging, from the molecular (telomere length and DNA methylation ages), to the physiological (physiological ages), and functional levels (cognitive score, functional age index, and frailty index).

### Telomere length

Relative telomere length (RTL) was measured from blood leukocytes as a T/S ratio by comparing the telomere sequence copy number in each participant’s (T) against a single-copy reference gene from β-hemoglobin (S) [26]. The T/S ratios were scaled to 10%.

### DNA methylation ages

Genome-wide methylation levels were assessed from leukocytes using Illumina’s Infinium HumanMethylation 450K BeadChip. Previous studies have developed various algorithms to calculate DNAmAges by aggregating the weighted averages of methylation level at specific GpG sites identified in a prediction model of CA and/or aging-related phenotypes. However, measurements of individual CpGs could be rather unreliable due to technical noise [27]. Herein. we adopted an advanced approach to construct principal-component version of DNAmAge (PCDNAmAges), which involves performing principal component analysis (PCA) on the CpG-level data and using PCs as input to predict various DNAmAges. Compared with traditional DNAmAges, PCDNAmAges have been shown to minimize random noise from individual CpGs and produce more stable trajectories in longitudinal measurements [28]. In this study, we included six PCDNAmAges that were trained on cross-sectional measurements of aging-related phenotypes, which were PCHorvathAge, PCSkin&bloodAge, PCHannumAge, PCPhenoAge, PCDNAmTL, and PCGrimAge. We additionally included DunedinPACE which is a novel DNAmAge trained on the longitudinal measures of 19 biomarkers that tracks organ-system integrity [29]. See more information regarding these DNAmAges in Supplementary Table 1.

### Physiological age

The physiological age (PhysioAge) in SATSA was constructed using the biomarkers assessed from immediate blood test, blood test in lab, urine strip test, and physical examination data [30]. A subsample in SATSA consisting of one random measurement for each participant was used to select biomarkers in correlation with CA (Pearson correlation > 0.1), which resulted in nine and five CA-correlated biomarkers for men and women respectively. Then, a single physiological age score was calculated by aggregating the loadings of these biomarkers and CA. The details of computation can be found elsewhere [30].

### Cognitive ability

Four cognitive domains, including verbal, spatial, memory, and processing speed abilities, were assessed in IPTs [31]. Scores on all measures were recoded into percentage of the total possible points for each test. A PCA-based approach was applied to derive an overall cognitive ability score. Namely, component scoring coefficients from the first component extracted at IPT1, excluding individuals with dementia, were used to construct a cognitive function measure at each IPT using test scores standardized to the mean and SD of each test at IPT1. *T-*score scaling (M = 50, SD = 10) was then applied to the components [30].

### Functional age index

Functional age index (FAI) was constructed based on four types of specific functional measures [7], including sensory abilities (vision and hearing) that were self-reported, and muscle strength, gait speed, and lung function that were measured by trained nurses. The four measures were standardized respectively based on the values from IPT2 and then summed to compose a general FAI.

### Frailty index

The Rockwood frailty index (FI) was conceptualized as a multidimensional indicator of vulnerability to a range of age-related adverse outcomes. In SATSA, FI was calculated as a ratio of the number of deficits present in a participant to a total of 42 health deficits. The 42 health deficits in SATSA were self-reported and covered various health outcomes, such as symptoms, diseases, disability, mood, and activities in daily living [32]. The FI ratios were scaled to 100%.

### Drug classifications and other covariates

Information regarding medication use was collected by questionnaires asking the participants to provide the names of drugs they were recently using. The Anatomical Therapeutic Chemical (ATC) classification system was applied to codify the self-reported drug names. At the therapeutic level, we categorized antihypertensive (ATC codes: C02, C03, C07, C08, and C09), lipid-lowering (C10A), and anti-diabetic (A10) drugs according to the first two or three level of ATC codes. Within these three major categories, we identified five subcategories with ≥ 50 ever-users, including diuretics (C03, *n* = 234), calcium channel blockers (CCBs) (C08C, C08D, C08E, *n* = 137), agents acting on the renin-angiotensin system (RAS) (C09A, C09C, *n* = 172), beta blockers (C07A, *n* = 240), and statin (C10AA, *n* = 125), with exclusion of drugs belonging to these subcategories but containing multiple active substances. Medication use status for each drug category was defined as a binary variable, indicating use/no use of any drugs within the category during each IPT.

Because medication use data in SATSA were self-reported, we validated the data by comparing reported usage with the information from the Swedish Prescribed Drug Registry (SPDR). SPDR is a national register established in July 2005 and collects all the prescribed drugs dispensed at pharmacies in Sweden (coded in ATC with dispensation date) [33]. We extracted the self-reported medication data in SATSA since 2005 (including IPT7-10) and retrieved the drugs purchased by SATSA participants within 1 year before the IPT date from SPDR. In comparison with purchase information from SPDR, 98–100% of the SATSA participants who reported using the drugs purchased the corresponding drugs within 1 year before the IPT data, while 88–99% of the participants who did not report using the drugs did not purchase the drugs (Supplementary Table 2).

Chronological age was ascertained by linking SATSA to the Swedish Population Registry. Smoking and fasting status were self-reported and classified as binary (yes/no). The number of medications currently used was counted as the number of different drugs identified at the fifth ATC level. Body mass index (BMI, kg/m^2^), systolic blood pressure (SBP, mmHg), and diastolic blood pressure (DBP, mmHg) were obtained from the physical examination at IPTs. Blood glucose level (mmol/L) and apoB/apoA ratio were obtained from lab testing of blood samples. Hypertension was defined as SBP > 140 mmHg or DBP > 90 mmHg or taking any antihypertensive drugs. For each individual, missing values for BMI were imputed with the average of the available measurements, while missing values for smoking status were imputed with the most recent available value.

### Statistical analysis

We first categorized the participants according to use of any antihypertensive, anti-diabetic, and lipid-lowering drugs during the follow-up (ever-user/never-user) or at the first available measurement (user/non-user). We described the characteristics within these subgroups and examined the differences using *t*-tests for qualitative factors and chi-square tests for categorical factors. To illustrate the relationship between different BA biomarkers, we obtained the BA residuals by regressing out the CA-related effects in a linear mixed model with fixed effects of CA and sex and random effects of individuals and twin pairs. Then, the correlations between BA residuals were calculated using a Pearson method with control for repeated measurements [34]. To examine the correlation patterns between uses of multiple drugs, we quantified the association between pairs of drug categories by fitting generalized equation estimation (GEE) models with one drug category as exposure and the other as outcome [35]. In these GEE models, we used logit link functions, thereby estimating the odds ratio relating the two medications.

We used conditional generalized estimating equations (cGEE) for estimating and testing the associations between drugs and BA biomarkers. When conditioning on the individual, cGEE includes an individual-specific intercept to model the effects of individual-constant factors and provides estimates that are protected against bias due to individual-constant confounders [36]. For each BA biomarker, a cGEE model was constructed with medication use as exposure and BA biomarker as outcome, conditioning on the individual to control for individual-constant confounders and adjusting for important individual-varying factors including CA, BMI, smoking status, and number of medications being currently used. In these cGEE models, we used identity link functions, thereby estimating the difference in mean BA biomarker (beta-coefficient) between users and non-users of the medication. To account for uses of multiple drugs, we included the three major drug categories (antihypertensive, antidiabetic, and lipid-lowering drugs) or their subcategories (diuretics, CCBs, beta blockers, agents acting on RAS, statins, insulin analogues, and non-insulin antidiabetic drugs) in a multivariable model. Because medication users necessarily had the relevant disorders to obtain the prescription, it is important to consider the possible confounding from participants’ disease status. We therefore included blood biomarkers underlying the indication disorders (systolic blood pressure (SBP) for hypertension, apoB/apoA ratio for hyperlipidemia, blood glucose level for diabetes, and fasting status) into the multivariable model.

We conducted a sensitivity analysis for the four antihypertensive drug subcategories using a subset of participants who had hypertension or developed hypertension during the follow-up. We included measurements since their first detectable hypertension. Because hypertension is considered an incurable chronic disorder, we assumed that these participants would live with hypertension afterwards. This analysis is expected to provide estimation independent of hypertension by comparing the change in BA biomarkers after hypertension onset. To demonstrate the presence of confounding by individual-constant factors, we repeated the analyses for the three major drug categories using a GEE model that does not condition on the individual. Possible selection bias could arise from our exclusion of the participants who only contributed with one IPT measurement. Therefore, we described the baseline characteristics of 12 BA biomarkers and medication use of participants who participated in one IPT against those who participated more. We also estimated the odds ratio for baseline BA biomarkers and medication use predicting the participation in IPTs with adjustment for CA using logistical regression. All the analyses were conducted in R 4.0.5, and the cGEE models were fitted using the drgee package [36]. To account for the relatedness between twin pairs in our data, all the confidence intervals (CIs) were constructed by bootstrapping that involved random resampling of pairs of twins 10,000 times (two-sided with *P* < 0.05). More details about cGEE models and bootstrapping are described in Supplementary Methods.

## Results

### Characteristics of participants in SATSA

As shown in Table 1, a total of 672 participants were included in the final analysis. At the first available measurement, 189 participants were using antihypertensive, antidiabetic, or lipid-lowering drugs. The category with most users was antihypertensive drugs (*n* = 179). The 189 medication users were older (*P* < 0.001) and had higher measures of FI score (*P* < 0.001), FAI (*P* = 0.0015), PCHorvathAge (*P* = 0.022), PCSkin&bloodAge (*P* = 0.029), PCHannumAge (*P* = 0.012), PCPhenoAge (*P* = 0.0069), PhysioAge (*P* < 0.001), and lower cognitive scores (*P* = 0.049). Moreover, the medication users tended to have higher BMI (*P* < 0.001); smoke less (*P* = 0.012); use more medications overall (*P* < 0.001); and have higher blood pressure (*P* for SBP < 0.001; *P* for DBP = 0.0028), lipids (apoB/apoA: *P* = 0.048), and blood glucose (*P* < 0.001). During follow-up, 410 individuals ever used any antihypertensive drugs, followed by lipid-lowering drugs (*n* = 132) and anti-diabetic drugs (*n* = 63); meanwhile, the ever-users were likely to participate in more IPTs (*P* < 0.001).

**Table 1:** Characteristics of participants and the uses of antihypertensive, anti-diabetic, and lipid-lowering drugs. The bold font indicates that the corresponding *P*-value is less than 0.05

Characteristics	At baseline (first available) IPT measurements		*P*
	Users (*n *= 189)	Non-users (*n *= 483)	
Drug categories (*n*)			
Antihypertensive drugs	179		
Anti-diabetic drugs	10		
Lipid lowering drugs	15		
Age (mean, SD)	66.62 (7.55)	62.78 (7.86)	**< 0.001**
Frailty index (ratio, %)	12.71 (8.25)	9.44 (6.96)	**< 0.001**
Cognitive score	50.87 (10.52)	52.65 (10.16)	**0.049**
Functional age index	51.30 (11.53)	48.05 (11.68)	**0.0015**
Physiological age (year)	69.48 (8.92)	63.63 (9.42)	**< 0.001**
Relative telomere length (ratio, 10%)	7.52 (2.84)	7.36 (2.12)	0.53
PCHorvathAge (year)	62.49 (8.34)	60.45 (9.08)	**0.022**
PCSkin&bloodAge (year)	58.89 (7.97)	57.02 (8.81)	**0.029**
PCHannumAge (year)	66.07 (8.02)	63.89 (8.84)	**0.012**
PCGrimAge (year)	78.67 (6.55)	77.79 (7.19)	0.21
PCPhenoAge (year)	64.49 (7.44)	62.27 (8.85)	**0.0069**
DunedinPACE (year)	1.07 (0.15)	1.05 (0.15)	0.12
PCDNAmTL (kilobase)	6.87 (0.18)	6.89 (0.19)	0.29
BMI	27.09 (4.22)	25.21 (3.58)	**< 0.001**
Currently smoking (n, %)	24 (13)	102 (21)	**0.012**
Number of drugs being used currently	3.16 (1.84)	1.39 (1.53)	**< 0.001**
SBP (mmHg)	161.36 (23.30)	148.57 (22.22)	**< 0.001**
DBP (mmHg)	89.15 (11.70)	86.31 (9.72)	**0.0034**
apoB/apoA ratio	1.06 (0.35)	1.00 (0.30)	**0.048**
Blood glucose (mmol/L)	5.46 (2.20)	4.69 (1.31)	**< 0.001**
Fasting before taking blood sample (*n*, %)	84 (44)	202 (42)	0.54
	During follow-up		
	Ever users(*n* **= **445)	Never users(*n* **= **227)	
Drug categories (*n*)			
Antihypertensive drugs	410		
Anti-diabetic drugs	63		
Lipid lowering drugs	132		
Number of IPT measurements	1964	782	
Mean number of IPT measurements (mean, SD)	4.41 (1.74)	3.44 (1.50)	**< 0.001**
Women (*n*, %)	268 (60)	127 (56)	0.29

### Correlation between biomarkers of biological aging and patterns of medication uses

The correlations between the 12 BA biomarkers after ruling out the effects from CA are presented in Supplementary Fig. 1. Generally, two correlation clusters were observed for six PCDNAmAges and for three functional age predictors, respectively. The six PCDNAmAges were generally correlated with each other (*P* < 0.05/78, corrected for 78 pairs of BA biomarkers), with coefficients ranging from 0.33 to 0.92 (absolute values); meanwhile, the three functional age predictors (FI, cognitive score, and FAI) were correlated with each other to a lesser extent (absolute values of coefficients ranging from 0.18 to 0.30, *P* < 0.05/78). Additionally, DunedinPACE was correlated with PCGrimAge (*r* = 0.20) and PCPhenoAge (*r* = 0.42). No distinct correlation patterns for other pairs of BAs were observed. When investigating the relationship between uses of multiple medications (Supplementary Figure 2), we observed strong associations either between antihypertensive, anti-diabetic, and lipid-lowering drug categories or between the seven subcategories (20 correlations with *P* < 0.05/24, corrected for 24 pairs of drug categories).

### Associations between medication use and biological ages

**Fig. 2:**
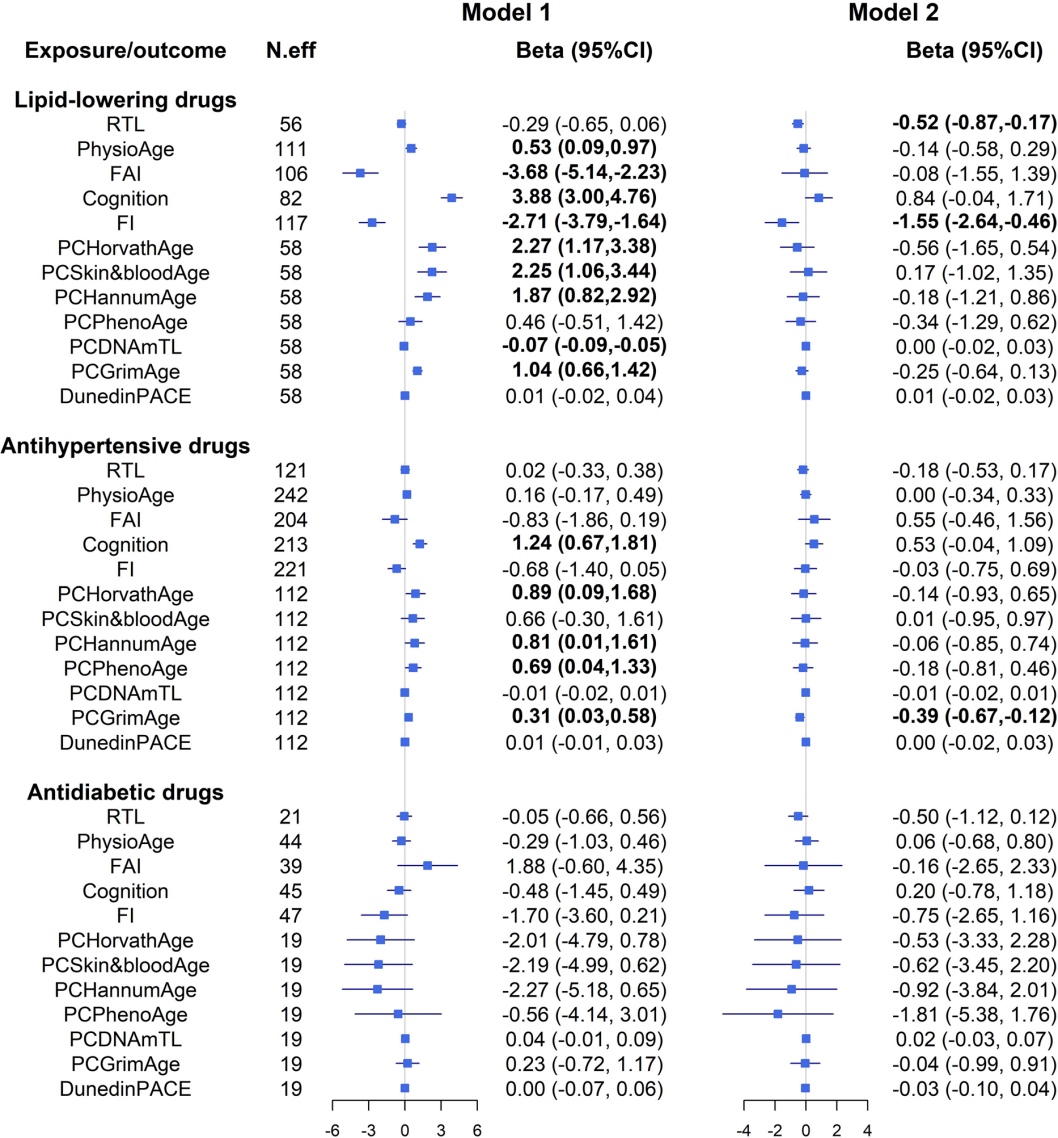
Associations between uses of antihypertensive, anti-diabetic, and lipid-lowering drugs and biomarkers of biological aging. The associations were estimated using cGEE model. All the models were adjusted for within-individual factors, including body mass index, currently smoking, age, number of drugs being currently used, SBP, apoB/apoA ratio, blood glucose level, fasting status, and three medication use variables (antihypertensive drugs, antidiabetic drugs, and lipid-lowering drugs). Model 1 is not conditioning on individuals, while model 2 is conditioning on individuals to additionally control for effects from individual-constant factors. N.eff stands for effective sample size, which is the number of participants who at some point used the medication during follow-up, therefore contributed to the estimation of drug effects. A bold font indicates that the corresponding *P*-value is less than 0.05. RTL, relative telomere length; PhysioAge, physiological age; FAI, functional age index; FI, frailty index

We first assessed the association between antihypertensive, anti-diabetic, or lipid-lowering drugs and 12 BAs. Model 1 is adjusted for important within-individual covariates including BMI, smoking status, age, number of drugs, SBP, apoB/apoA ratio, blood glucose level, and fasting status, while model 2 is further conditioning on the individual to adjust for or individual-constant factors. As shown in Fig. 2, the estimates changed markedly in model 2 after taking individual-constant factors into account, which indicated substantial bias from individual-constant factors. In model 2, taking lipid-lowering drugs was associated with shortening of TL (RTL: beta = − 0.52, 95%CI = − 0.87 to − 0.17) but reduced frailty (FI: beta = − 1.55, 95%CI =− 2.64 to − 0.46), while taking antihypertensive drugs was associated with a reduced PCGrimAge (beta = − 0.39, 95%CI = − 0.67 to − 0.12). No associations between taking antidiabetic drugs and any BA biomarkers were observed, possibly due to the limited size of ever-users.

We subsequently analyzed five drug subcategories with at least 50 ever-users (Table 2). The point estimates for CCBs indicated a decrease in seven DNAmAges (a decrease of 0.57 to 1.74 years in five PCDNAmAges, a 0.03-year decrease of aging rate in DunedinPACE, and an elongation of 0.01 in PCDNAmTL), among which the estimates for PCHorvathAge (beta = − 1.28, 95%CI = − 2.34 to − 0.21), PCSkin&bloodAge (beta = − 1.34, 95%CI = − 2.61 to − 0.07), PCPhenoAge (beta = − 1.74, 95%CI = − 2.58 to − 0.89), and PCGrimAge (beta = − 0.57, 95%CI = − 0.96 to − 0.17) excluded the null. In addition to DNAmAges, CCBs was also associated with decreased functional biological ages, shown in FAI (beta = − 2.18, 95%CI = − 3.65 to − 0.71) and FI (beta = − 1.31, 95%CI = − 2.43 to − 0.18). Among other antihypertensive drugs, uses of diuretics, beta blockers, and agents acting on RAS were associated with an increased cognitive score (diuretics: beta = 0.94, 95%CI = 0.31 to 1.58; beta blockers: beta = 0.63, 95%CI = 0.01 to 1.25; agents acting on RAS: beta = 0.73, 95%CI = 0.04 to 1.42), despite their adverse associations with other BA biomarkers, such as FAI (beta blockers: beta = 1.52, 95%CI = 0.35 to 2.68), PCHorvathAge (diuretics: beta = 1.06, 95%CI = 0.10 to 2.01), PCSkin&bloodAge (diuretics: beta = 1.26, 95%CI = 0.09 to 2.42; agents acting on RAS: beta = 1.58, 95%CI = 0.26 to 2.90), PCHannumAge (agents acting on RAS: beta = 1.36, 95%CI = 0.02 to 2.69), and PCGrimAge (agents acting on RAS: beta = 0.61, 95%CI = 0.16 to 1.06). Among lipid-lowering drugs, statin use was associated with decreased FI (beta = -1.08, 95%CI = − 2.01 to − 0.15).

**Table 2:** Associations between uses of subcategories within antihypertensive and lipid-lowering drugs and biomarkers of biological aging. The associations were estimated using cGEE models with adjustment for within-individual factors, including body mass index, currently smoking, age, number of medications being currently used, SBP, apoB/apoA ratio, blood glucose level, fasting status, and seven medication use variables (diuretics, calcium channel blockers, beta blockers, agents acting on renin-angiotensin system, statins, insulin and analogues, and non-insulin anti-diabetics). All the models were additionally conditioning on individuals to control for effects from individual-constant factors. N.eff stands for effective sample size, which is the number of participants who at some point used the medication during follow-up, therefore contributed to the estimation of drug effects. A bold font indicates that the corresponding *P*-value is less than 0.05. RTL, relative telomere length; PhysioAge, physiological age; FAI, functional age index; FI, frailty index

BA biomarkers	Diuretics		Beta blockers		Calcium channel blockers		Agents acting on RAS		Statins	
	N.eff	Beta (95%CI)	N.eff	Beta (95%CI)	N.eff	Beta (95%CI)	N.eff	Beta (95%CI)	N.eff	Beta (95%CI)
RTL	77	0.02 (− 0.41, 0.46)	85	− 0.21 (− 0.58, 0.15)	63	− 0.11 (− 0.46, 0.24)	68	0.02 (− 0.37, 0.42)	54	− 0.38 (− 0.78, 0.01)
PhysioAge	163	− 0.05 (− 0.42, 0.31)	169	0.01 (− 0.37, 0.38)	112	− 0.20 (− 0.58, 0.18)	147	0.02 (− 0.35, 0.39)	106	0.02 (− 0.40, 0.43)
FAI	141	0.19 (− 1.00, 1.37)	136	**1.52 (0.35, 2.68)**	102	− **2.18 (**− **3.37,** − **0.99)**	129	0.94 (− 0.32, 2.19)	105	0.25 (− 1.18, 1.68)
Cognition	132	**0.94 (0.31, 1.58)**	152	**0.63 (0.01, 1.25)**	95	− 0.18 (− 0.91, 0.54)	109	**0.73 (0.04, 1.42)**	76	0.81 (− 0.06, 1.68)
FI	154	− 0.30 (− 1.23, 0.63)	151	**0.91 (0.02, 1.79)**	106	− **1.31 (**− **2.43,** − **0.18)**	135	− 0.67 (− 1.61, 0.27)	112	− **1.08 (**− **2.01,** − **0.15)**
PCHorvathAge	71	**1.06 (0.10, 2.01)**	76	− 0.20 (− 0.90, 0.50)	61	− **1.28 (**− **2.34,** − **0.21)**	65	1.13 (− 0.15, 2.42)	55	− 0.45 (− 1.44, 0.54)
PCSkin&bloodAge	71	**1.26 (0.09, 2.42)**	76	− 0.17 (− 1.08, 0.74)	61	− **1.34 (**− **2.61,** − **0.07)**	65	**1.58 (0.26, 2.90)**	55	0.33 (− 0.76, 1.42)
PCHannumAge	71	0.42 (− 0.60, 1.43)	76	− 0.44 (− 1.22, 0.33)	61	− 0.98 (− 2.06, 0.11)	65	**1.36 (0.02, 2.69)**	55	− 0.22 (− 1.20, 0.76)
PCPhenoAge	71	0.10 (− 0.61, 0.81)	76	0.08 (− 0.57, 0.74)	61	− **1.74 (**− **2.58,** − **0.89)**	65	0.89 (− 0.51, 2.28)	55	− 0.31 (− 1.28, 0.65)
PCDNAmTL	71	− 0.01 (− 0.03, 0.01)	76	− 0.01 (− 0.02, 0.01)	61	0.01 (− 0.01, 0.02)	65	− 0.02 (− 0.04, 0.00)	55	0.01 (− 0.01, 0.03)
PCGrimAge	71	− 0.04 (− 0.38, 0.30)	76	− 0.21 (− 0.53, 0.12)	61	− **0.57 (**− **0.96,** − **0.17)**	65	**0.61 (0.16, 1.06)**	55	− 0.19 (− 0.60, 0.21)
DunedinPACE	71	0.00 (− 0.02, 0.03)	76	0.01 (− 0.01, 0.04)	61	− 0.03 (− 0.06, 0.00)	65	− 0.04 (− 0.08, 0.00)	55	0.01 (− 0.02, 0.05)

In the sensitivity analysis performed in a subset of participants who had hypertension, the associations observed for four antihypertensive drugs were largely unchanged (Supplementary Table 3). In the sensitivity analysis testing the potential selection bias by excluding participants who only participated in one IPT (Supplementary Table 4), only baseline CA and three functional BA biomarkers (FAI, cognition, and FI) differed significantly between participants who participated in one IPT and who participated in more IPTs (*P* values ranging from < 0.001 to 0.85). Similarly, in the regression model with adjustment for CA, only three functional BA biomarkers at baseline (FAI, cognition, and FI) predicted the future participation in IPTs (*P* values ranging from 0.002 to 0.01), while other BA biomarkers as well as all medication uses showed no associations (all *P* values > 0.05).

## Discussion

In this study, we observed potential georoprotective effects, indexed by lower biological aging measures at the epigenetic and/or functional level, particularly for calcium channel blockers (CCBs) and weaker findings for antihypertensive and lipid lowering medications. Specifically, we found that treatment with antihypertensive drugs was associated with lower GrimAge. When considering drug subtypes, the use of CCBs consistently corresponded to a decrease in DNAmAges, particularly for PCGrimAge (a 0.57-year decrease), PCSkin&bloodAge (a 1.34-year decrease), PCPhenoAge (a 1.74-year decrease), and PCHorvathAge (a 1.28-year decrease), as well as a 2.81-year decrease in FAI and a decrease of 1.31 in FI, revealing a putative geroprotective effect by CCBs at molecular and functional level. The results for other drug subtypes were however inconsistent. Uses of diuretics, beta blockers, and agents acting on RAS were associated with a higher cognitive ability while yielding adverse associations with DNAmAges and FAI. Statin use was associated with decreased FI, but no associations with other BA biomarkers was observed.

To our knowledge, few studies have investigated the relationship between medication use and biological aging. Two studies have examined associations between medication use and DNAmAge accelerations (residuals for DNAmAges regressing on CA, similar to our results after adjusting CA in the model) based on longitudinal data examined over two visits. In the Genetic Epidemiology Network of Arteriopathy (GENOA) study consisting of 226 American Africans, the participants who started antihypertensive drugs in the second visit had a 0.96-year lower GrimAge acceleration as compared with those who never took any antihypertensive drugs in either visit [21]. Meanwhile, similar to our finding of CCBs in association with decreased DNAmAges, the GENOA study observed that initiation of CCBs was negatively associated with PhenoAge acceleration (beta = − 1.40, *P* = 0.089). However, in the Normative Aging Study (NAS) comprising 546 older white men, initiation of CCBs was associated with increased DNAmAge acceleration (HorvathAge: beta = 0.41, *P* = 0.062) [22]. The present study differs from these two studies in several important aspects, including the population age (mean age at baseline in SATSA, 67.9 years old; GENOVA, 54.0; NAS, 71.6) and ancestry (SATSA and NAS, European ancestry; GENOVA, African ancestry), sex composition (SATSA and GENOVA, men and women; NAS, men), number of measurements (mean in SATSA, 2.6; GENOA and NAS, 2), follow-up duration (mean in SATSA, 13.7 years; GENOA, 5.4; NAS, 3.86), DNAmAge measures (SATSA, six PCDNAmAges; GENOA and NAS, up to four traditional DNAmAges), and statistical models (SATSA, cGEE model adjusting for unmeasured individual-constant factors; GENOA and NAS, linear mixed model adjusting for measured base-line factors). Overall, compared with these two studies, the present study provided novel evidence supporting the epigenetic effects of CCBs on aging under the context of advanced measures of multiple DNAmAges (six PCDNAmAges), additional longitudinal measurements, and longer follow-up duration.

The effects of CCBs on functional capabilities or frailty remains poorly understood. A cross-sectional study assessed frailty prevalence among 138 individuals aged 60 to 90 years old and found that CCB use was associated with a 70% lower risk for frailty (95%CI = 0.09 to 0.77) [37]. However, in a cohort study that followed 1394 participants aged ≥ 70 years up for 5 years, CCB use at baseline was not associated with frailty (HR = 1.10, 95%CI = 0.76 to 1.60) [38]. Of note, frailty in the prior two studies was defined using five physical components, including weight loss, exhaustion, low physical activity, slow walking speed, and weakness, which differed from FI defined in the present study that incorporated 42 health deficits including physical abilities, diseases, and mood. Indeed, CCBs have been shown to reduce the risk of developing age-related diseases that severely impair functional capabilities, such as stroke and neurodegenerative diseases [39–43]. In particular, several large retrospective studies have found that the use of CCBs was associated with a lower risk of developing Parkinson’s disease [41–43]. The potential pathways might be through CCBs blocking neuronal voltage-gated L-type calcium channels to rejuvenate substantia nigra dopamine neurons that are critical in psychomotor functions [44]. Overall, these mechanisms might benefit functional capabilities in old age and link CCBs to a slowed functional biological age as observed in the current study.

In this study, the decreasing pattern of DNAmAges and functional biological ages associated with CCBs is distinct from the results for other antihypertensive drug subtypes. This might imply that these observed effects are specific to CCBs and not a result of the common action of lowering blood pressure. In fact, previous evidence have revealed that high intracellular Ca2+ concentration promotes cellular senescence and death [45]. CCBs, through their regulation of Ca2+ levels, have been found to trigger AMP-activated protein kinase signaling and increase autophagic flux, thereby suppressing cellular senescence [46]. On an organismal level, verapamil, an L-type CCB, has also been found to extend lifespan and improve age-related physiological parameters, such as locomotion, trashing, and thrashing, in *C. elegans.* Furthermore, this study examined the life-extending mechanisms and proposed that verapamil extended *C. elegans* lifespan by modulating intracellular Ca2+ concentration, which subsequently inhibited calcineurin activity and activated autophagy [18].

A notable advantage of this study is the longitudinal study design allowing the use of a cGEE model conditioning on individuals. This analysis estimated the drug effect by comparing change of BA biomarkers within the same individual when taking a drug or not. As a result, we were able to control for unmeasured individual-constant confounders and provide more reliable estimation of drug effects. Second, we included 12 BA biomarkers from the molecular to the functional level, which allows this study to capture the influence of medication use on different aspects of biological aging. Moreover, instead of traditional DNAmAges calculated on individuals CpGs, we used the newly developed PCDNAmAges, which reduce the random noise from individual CpGs and produce more stable DNAmAge measures across longitudinal measurements. Third, considering that the medication use data in SATSA were self-reported and could result in misclassification, we performed validation using information from the national register database and found a high consistency rate between the two data sources for the drugs analyzed in this study. Finally, the mean follow-up duration among the participants included in this study is 13.7 years, which is long enough to capture the change of BA biomarkers caused by medication use.

This study also has some limitations. First, participants who contributed to only one IPT measurement were excluded from the analysis, which might lead to selection bias when medication uses and biological age both affect the drop-out. However, the results from our sensitivity analysis showed that use of antihypertensive, antidiabetic, or lipid-lowering drugs and a majority of the BA biomarkers (except for FAI, cognition, and FI) were not associated with the participants’ participation in more than one IPT. Second, since antihypertensive, antidiabetic, and lipid-lowering drugs were usually used for the treatment of hypertension, diabetes, and hyperlipidemia, our results might indeed capture the effects of these disorders on aging (indication bias). However, these disorders are usually thought to accelerate aging [47], which would drive the association estimates towards the null or adverse direction, in contrast to our findings of geroprotective effects for CCBs. Moreover, we adjusted for biomarkers underlying the continuum of these disorders in all models and performed sensitivity analysis for antihypertensive drugs by comparing the change of BA biomarkers after hypertension onset. Unfortunately, regarding the drugs demonstrating adverse associations with aging in this study, it remains difficult to ascertain whether the associations are due to drug effects or indication bias. Third, participants might modify their lifestyle after being diagnosed with hypertension, diabetes, or hyperlipidemia, which would likely affect biological aging and confound the association between medication uses and BA biomarkers. In our analysis, we adjusted for smoking status as an indicator for the change of lifestyles. Indeed, regular habits to control these disorders after diagnosis, such as low-carb diet for diabetes, could be considered approximately individual-constant factors, thus being controlled for in our cGEE models to some extent. Finally, the generalizability of this study could be limited to the older Swedish population. Also, the results for other drug subtypes except CCBs were inconsistent, and further studies with larger sample size are warranted to reexamine our results.

In conclusion, our results suggest that CCBs may hamper biological aging captured by the BA biomarkers measured at epigenetic and/or functional level and future intervention studies are warranted to confirm our findings.

## Supplementary information

ESM 1 (DOCX 15380 kb)
