# Supplementary material for: Longitudinal associations between use of antihypertensive, antidiabetic, and lipid-lowering medications and biological aging
Source: GeroScience. 2023 Apr 10;45(3):2065–78. doi: 10.1007/s11357-023-00784-8 (PMC10400489; doi:10.1007/s11357-023-00784-8)

**Supplementary Methods**

1. **Conditional generalized estimating equation model**

We let $n_{i}$denote the $i$th cluster and $n_{j}$denote $j$th individual within cluster $i$. We have observations for individual within each cluster regarding an outcome ($Y_{ij}$), an exposure ($X_{ij}$), and a vector of covariates ($V_{ij}$). We also have covariates which are constant within clusters ($W_{i}$). Like most methods for within-cluster analysis, there are two main assumptions:

**Assumption 1**: the clusters are independent

($Y_{1}, X_{1}, V_{1}, W_{1}$), … , $(Y_{m},X_{m},V_{m},W_{m})$ are independent

**Assumption 2**: the observations within each cluster are conditionally independent given the cluster-constant covariates

($Y_{i1}, X_{i1}, V_{i1}$), … , $\left( Y_{ij},X_{ij},V_{ij} \right)$ are conditionally independent, given $W_{i}$

Then the conditional generalized estimating equation (cGEE) model is considered as the form

$$g\left\{ E(Y_{ij}|X_{ij}, V_{ij}, W_{i}) \right\}=a_{i}+\beta X_{ij}+\gamma V_{ij}$$

where $g(.)$ is either the identity link or the log link. In this model, the cluster-specific intercept $\alpha_{i}$ incorporates the cluster-constant covariates $W_{i}$. The target parameter $\beta$ quantifies the conditional association between within-cluster exposure $X_{ij}$ and outcome $Y_{ij}$ (1).

1. **cGEE models in this study**

An important concern in this longitudinal study is that the association between medication use and BA biomarker might be confounded by individual-constant factors, particularly when such factors are difficult to measure or quantify, e.g. genes and socioeconomic factors. Since all the participants in SATSA were above 50 years old, we assumed that factors, such as genes and socioeconomic factors (e.g. obtained education level and income), rarely changed at a late age and therefore were individual-constant. Therefore, we conditioned cGEE on the individual to control for individual-constant factors ($W_{i}$). Such individual-constant factors, even though unmeasured, were implicitly adjusted for in the cGEE model when conditioning on the individual. In addition, we also adjusted for important individual-varying factors ($V_{ij}$), such as chronological age, BMI, smoking status, number of medications being currently used, blood glucose level, apoB/apoA ratio, systolic blood pressure, and fasting status. Briefly, we fitted the model shown as below,

$$g\left\{ E({BA}_{ij}|{Med}_{ij}, V_{ij}, W_{i}) \right\}=a_{i}+\beta{MED}_{ij}+\gamma V_{ij}$$

where $g(.)$ is identity link. ${BA}_{ij}$ is the measure of biomarker of biological aging (BA biomarker), ${MED}_{ij}$ is a vector of three major drug classes (antihypertensive drugs, lipid-lowering drugs, and antidiabetic drugs) or seven drug subtypes (diuretics, calcium channel blockers, beta blockers, agents acting on renin-angiotensin system, statins, insulin and analogues, and non-insulin antidiabetics), $a_{i}$ absorbs the effects from individual-constant covariates $W_{i}$, and $V_{ij}$ are a vector of individual-varying covariates we fit in. Below is an illustration of cGEE model used in this study.

**Supplementary Method Figure 1. An illustration for cGEE model used in this study.** Each dot represents an observation with medication use and BA biomarker measure for respective individual during follow-up.


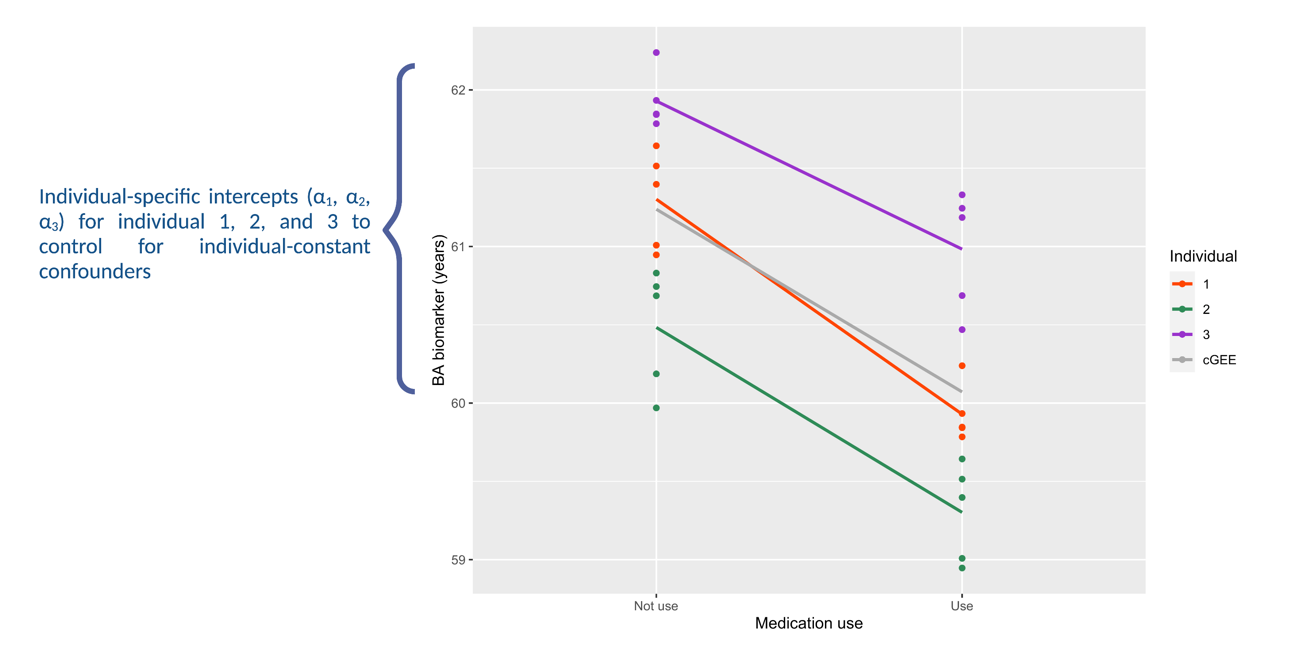


In this model, only individuals who changed the covariate (${MED}_{ij}$ or $V_{ij}$) could provide effective before-after contrast and substantially contribute to the estimation of effects by the covariate. For example, only participants who at some points during follow up used medication would contribute to the estimation of the drug effect on the BA biomarker level. Participants who always or never used mediation during follow up would not be included in the estimation of the drug effect on the BA biomarker level. However, they could experience change in covariates (e.g. BMI and chronological age), subsequently contributing to the estimation of the effects by these covariates, and thereby improving the precision of the whole model. Herein we counted the number of participants who at some point used medication in our data and showed this number as effective size (N.eff) in our results.

The SATSA data has two level of clustering, namely, at individuals (longitudinal measurements) and at twin pairs (twinship). When conditioning on the individual, the within-twin-pair correlation could violate the model assumption 1, that is, the clusters should be independent. Violation of this assumption may bias the standard errors, but not the estimated effect sizes. To account for such within-twin-pair correlation, we applied bootstrapping to construct the corrected standard errors by resampling twin pairs with replacement for 10,000 times. Each resampling contained the same size of twin pairs as in the primary data, e.g. sampling 100 twin pairs with replacement out of 100 twin pairs. A beta coefficient was estimated using cGEE model from each collection of twin pairs throughout the resampling process. Finally, a standard error was computed for the 10,000 beta coefficients obtained through resampling and used as the corrected standard error for our primary effect size estimate.

**Reference**

1. Goetgeluk S, Vansteelandt S. Conditional Generalized Estimating Equations for the Analysis of Clustered and Longitudinal Data. Biometrics. 2008;64(3):772-80.

2. Zetterqvist J, Sjölander A. Doubly Robust Estimation with the R Package drgee. Epidemiologic Methods. 2015;4(1):69-86.

**Supplementary Table 1. Characteristics of DNA methylation ages used in the present study.** The information for traditional DNA methylation ages (DNAmAges) developed based on individual CpGs are presented in the table, including names, tissues for DNA methylation measure, phenotypes used in the training model, and the number of CpGs being identified. For the DNAmAges that are available with principal-component version (PCDNAmAges), we used the PCDNAmAges in our longitudinal analysis to minimize the influence from technical noise.

| **DNAmAge** | **Tissues for DNA methylation measure** | **Phenotypes used in the training model** | **No. of CpGs being identified** | **Source** | **Is principal-component version (PCDNAmAge) available?** |
| --- | --- | --- | --- | --- | --- |
| HorvathAge | Multiple tissues | Chronological age | 353 | (Horvath, 2013) | Yes |
| Skin&bloodAge | Skin, blood, and saliva | Chronological age | 391 | (Horvath et al., 2018) | Yes |
| HannumAge | Blood | Chronological age | 71 | (Hannum et al., 2013) | Yes |
| PhenoAge | Blood | Chronological age, albumin, creatinine, glucose, C-reactive protein levels, lymphocyte percentage, mean cell volume, red blood cell distribution width, alkaline phosphatase, white blood cell count, and chronological age | 513 | (Levine et al., 2018) | Yes |
| DNAmTL | Blood | Telomere length | 140 | (Lu, Seeboth, et al., 2019) | Yes |
| GrimAge | Blood | Adrenomedullin, beta-2 microglobulin, growth differentiation factor 15, Cystatin C, leptin, plasminogen activation inhibitor 1, and tissue inhibitor metalloproteinase 1, smoking pack-years, and chronological age | 1030 | (Lu, Quach, et al., 2019) | Yes |
| DunedinPACE | Blood | Body mass index (BMI), Waist-hip ratio, Glycated hemoglobin, Leptin, Blood pressure (mean arterial pressure), Cardiorespiratory fitness (VO_2_Max), Forced vital capacity ratio (FEV_1_/FVC), Forced expiratory volume in one second (FEV_1_), Total cholesterol, Triglycerides, High density lipoprotein (HDL), Lipoprotein(a), Apolipoprotein B100/A1 ratio, estimated Glomerular Filtration Rate (eGFR), Blood Urea Nitrogen (BUN), High Sensitivity C-reactive Protein (hs-CRP), White blood cell count, mean periodontal attachment loss (AL), and the number of dental-caries-affected tooth surfaces (tooth decay). | 173 | (Belsky et al., 2022) | No |

**Supplementary Table 2**. **Validation of self-reported medication use data from SATSA.** The validation was performed by comparing the self-reported medication use data in IPT7-10 from SATSA with the drug purchase data retrieved from the Swedish Prescribed Drug Register within one year before the IPT date.

| **Drug type /  Purchased the drug within one year before IPT date** | **Reported using the drug in SATSA** | |
| --- | --- | --- |
|  | **Yes** | **No** |
| **Antihypertensive drugs** |  |  |
| Yes (sensitivity, %) | 681 (0.99) | 61 |
| No (specificity, %) | 4 | 461 (0.88) |
| **Diuretics** |  |  |
| Yes (sensitivity, %) | 294 (1.00) | 52 |
| No (specificity, %) | 2 | 859 (0.94) |
| **Calcium channel blockers** |  |  |
| Yes (sensitivity, %) | 228 (0.99) | 36 |
| No (specificity, %) | 2 | 941 (0.96) |
| **Beta blockers** |  |  |
| Yes (sensitivity, %) | 362 (1.00) | 56 |
| No (specificity, %) | 1 | 788 (0.93) |
| **Agents acting on renin-angiotensin system** |  |  |
| Yes (sensitivity, %) | 329 (1.00) | 44 |
| No (specificity, %) | 0 | 834 (0.95) |
|  |  |  |
| **Antidiabetic drugs** |  |  |
| Yes (sensitivity, %) | 97 (0.98) | 8 |
| No (specificity, %) | 2 | 1100 (0.99) |
|  |  |  |
| **Lipid-lowering drugs** |  |  |
| Yes (sensitivity, %) | 305 (0.99) | 60 |
| No (specificity, %) | 4 | 838 (0.93) |
| **Statins** |  |  |
| Yes (sensitivity, %) | 295 (0.99) | 52 |
| No (specificity, %) | 2 | 858 (0.94) |

**Supplementary Table 3. Associations between use of subcategories within antihypertensive drugs and biomarkers of biological aging among the subgroup of participants with hypertension.** The analysis only contained measurements since first detectable hypertension in order to compare the change of BA biomarkers after hypertension onset. The associations were estimated using cGEE models with adjustment for individual-varying factors, including body mass index, currently smoking, age, number of medications being currently used, SBP, apoB/apoA ratio, blood glucose level, fasting status, and seven medication use variables (diuretics, calcium channel blockers, beta blockers, agents acting on renin-angiotensin system, statins, insulin and analogues, and non-insulin antidiabetics). All the models were also conditioning on the individual to control for effects from individual-constant factors. N.eff stands for effective sample size, which is the number of participants who at some point used the medication during follow-up, therefore contributed to the estimation of drug effects. BA biomarkers indicate biomarkers of biological aging; RTL, relative telomere length; PhysioAge, physiological age; FAI, functional age index; FI, frailty index.

| **BA biomarkers** | **Diuretics** | | **Beta blockers** | | **Calcium channel blockers** | | **Agents acting on RAS** | |
| --- | --- | --- | --- | --- | --- | --- | --- | --- |
|  | **N.eff** | **Beta (95%CI)** | **N.eff** | **Beta (95%CI)** | **N.eff** | **Beta (95%CI)** | **N.eff** | **Beta (95%CI)** |
| RTL | 74 | 0.05 (-0.39, 0.48) | 82 | -0.17 (-0.54, 0.20) | 60 | -0.09 (-0.44, 0.27) | 65 | 0.14 (-0.22, 0.50) |
| PhysioAge | 160 | -0.07 (-0.43, 0.30) | 160 | 0.05 (-0.32, 0.42) | 108 | -0.23 (-0.62, 0.16) | 144 | 0.08 (-0.29, 0.45) |
| FAI | 137 | 0.06 (-1.12, 1.24) | 128 | **1.37 (0.22, 2.51)** | 98 | **-2.65 (-3.87, -1.42)** | 126 | 0.31 (-0.96, 1.59) |
| Cognition | 130 | **1.14 (0.52, 1.76)** | 146 | **0.67 (0.04, 1.29)** | 92 | 0.15 (-0.55, 0.84) | 107 | **0.95 (0.25, 1.65)** |
| FI | 150 | -0.39 (-1.31, 0.53) | 143 | **1.03 (0.15, 1.92)** | 103 | **-1.61 (-2.69, -0.52)** | 133 | -0.95 (-1.91, 0.01) |
| PCHorvathAge | 69 | **0.99 (0.10, 1.87)** | 74 | -0.16 (-0.84, 0.51) | 59 | **-1.27 (-2.32, -0.22)** | 61 | 1.09 (-0.24, 2.43) |
| PCSkin&bloodAge | 69 | **1.19 (0.06, 2.32)** | 74 | -0.18 (-1.08, 0.72) | 59 | **-1.28 (-2.52, -0.04)** | 61 | **1.47 (0.09, 2.86)** |
| PCHannumAge | 69 | 0.40 (-0.59, 1.40) | 74 | -0.36 (-1.13, 0.40) | 59 | -0.95 (-2.03, 0.14) | 61 | 1.37 (-0.03, 2.77) |
| PCPhenoAge | 69 | -0.07 (-0.74, 0.61) | 74 | 0.11 (-0.53, 0.75) | 59 | **-1.84 (-2.66, -1.01)** | 61 | 1.00 (-0.46, 2.46) |
| PCDNAmTL | 69 | -0.01 (-0.03, 0.01) | 74 | -0.01 (-0.02, 0.01) | 59 | 0.00 (-0.01, 0.02) | 61 | -0.02 (-0.04, 0.01) |
| PCGrimAge | 69 | -0.19 (-0.52, 0.14) | 74 | -0.23 (-0.53, 0.08) | 59 | **-0.60 (-1.00, -0.19)** | 61 | **0.52 (0.04, 0.99)** |
| DunedinPACE | 69 | 0.00 (-0.02, 0.02) | 74 | 0.01 (-0.01, 0.03) | 59 | -0.03 (-0.07, 0.00) | 61 | -0.04 (-0.08, 0.00) |

**Supplementary Table 4. Characteristics of aging status and medication use between participants who attended one IPT *v.s.* more than one IPT at their baseline IPT measurement.** The baseline IPT measurement is the first available IPT measurement for each participant. The ORs were estimated in the logistic model with adjustment for age.

| **Characteristics** | **At the baseline IPT measurement** | | **P** | **Age-adjusted OR predicting the attendance of more than one IPT** | **P** |
| --- | --- | --- | --- | --- | --- |
|  | **Attended one IPT (n=156)** | **Attended more than one IPT (n=697)** |  |  |  |
| **Age (mean, SD)** | 68.55 (9.15) | 63.31 (8.16) | **<0.001** |  |  |
| **Relative telomere length (ratio, 10%)** | 7.40 (0.87) | 7.41 (1.35) | 0.98 | 0.99 (0.61,1.62) | 0.97 |
| **Physiological age (year)** | 69.81 (10.74) | 64.01 (9.38) | **<0.001** | 0.98 (0.93,1.03) | 0.51 |
| **Functional age index** | 49.86 (12.05) | 42.24 (10.59) | **0.01** | 0.94 (0.90,0.98) | **0.01** |
| **Cognition score** | 47.01 (10.69) | 51.89 (10.17) | **<0.001** | 1.03 (1.01,1.05) | **0.01** |
| **Frailty index (ratio, %)** | 14.29 (13.94) | 8.08 (5.93) | 0.06 | 0.92 (0.87,0.97) | **0.002** |
| **PCHorvathAge (year)** | 58.57 (6.24) | 57.98 (8.39) | 0.8 | 1.00 (0.91,1.10) | 0.98 |
| **PCSkin&bloodAge (year)** | 54.55 (5.45) | 55.06 (7.25) | 0.8 | 1.02 (0.92,1.13) | 0.69 |
| **PCHannumAge (year)** | 60.67 (5.07) | 61.08 (7.72) | 0.83 | 1.02 (0.93,1.13) | 0.64 |
| **PCPhenoAge (year)** | 57.06 (4.99) | 56.71 (7.11) | 0.85 | 1.02 (0.90,1.17) | 0.71 |
| **PCDNAmTL (kilobase)** | 6.98 (0.11) | 6.94 (0.19) | 0.30 | 0.13 (0.00,9.41) | 0.35 |
| **PCGrimAge (year)** | 73.07 (6.15) | 72.16 (6.05) | 0.68 | 1.02 (0.84,1.24) | 0.83 |
| **DunedinPACE (year)** | 1.00 (0.14) | 1.03 (0.14) | 0.59 | 4.83 (0.04,646.28) | 0.53 |
| **Medication use (n, %)** |  |  |  |  |  |
| Antihypertensive drugs | 39 (25.0) | 165 (23.7) | 0.80 | 1.20 (0.79,1.83) | 0.39 |
| Anti-diabetic drugs | 6 (3.8) | 11 (1.6) | 0.084 | 0.45 (0.16,1.29) | 0.14 |
| Lipid lowering drugs | 1 (0.6) | 13 (1.9) | 0.34 | 2.83 (0.36,22.24) | 0.32 |

**Supplementary Figure 1.** **Correlation between chronological age and 12 biomarkers of biological aging in SATSA.** The correlation coefficients were estimated using residuals for BA biomarkers after ruling out the effects by chronological age in a linear mixed model. * p<0.05, **p<0.05/78 pairs of CA and BA biomarkers. BA biomarkers indicates biomarkers of biological aging.


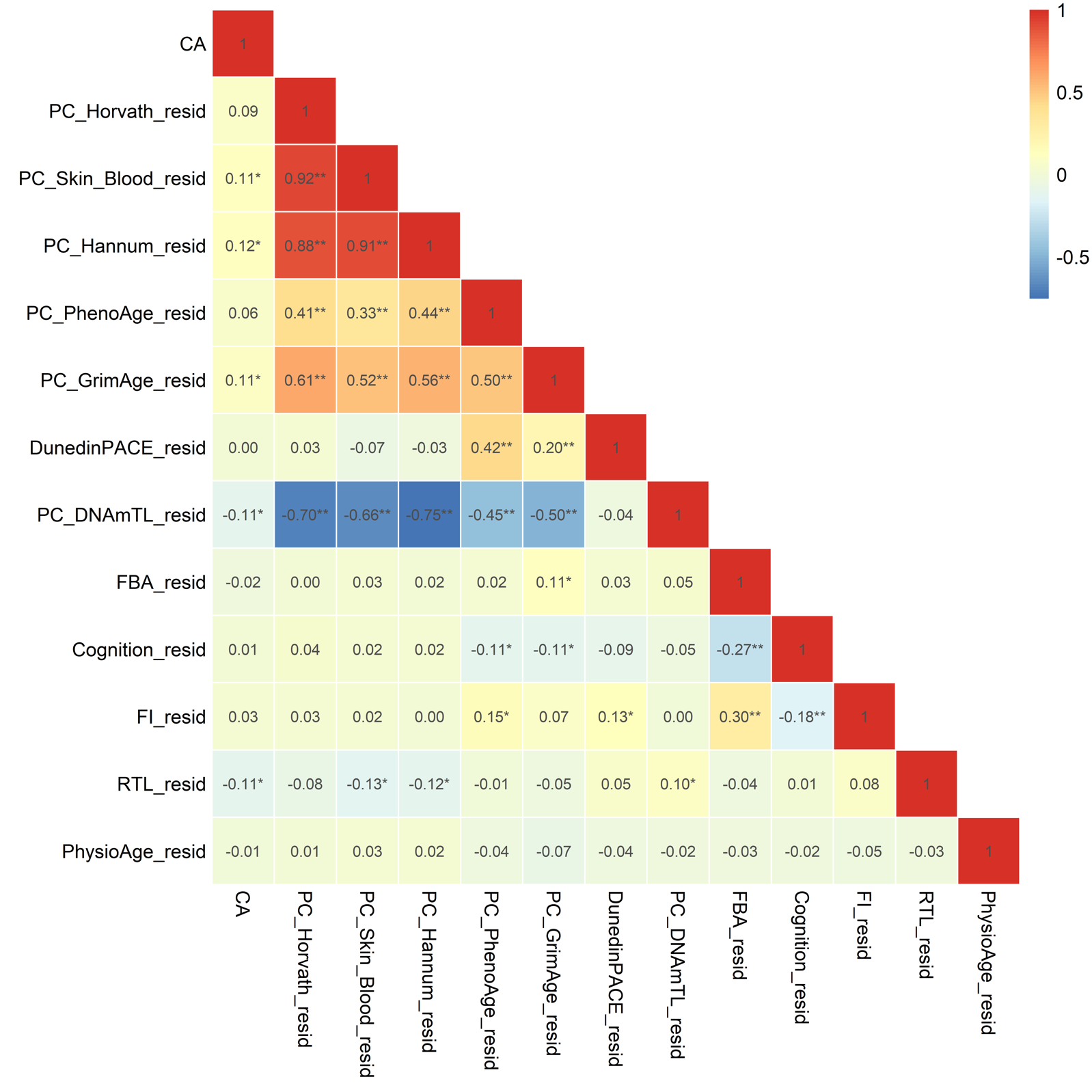


**Supplementary Figure 2.** **Relationship between medication use in SATSA.** The number in each cell was odds ratio for the association between pair of drugs estimated in GEE model. * p<0.05, ** p<0.05/24 pairs medication categories.


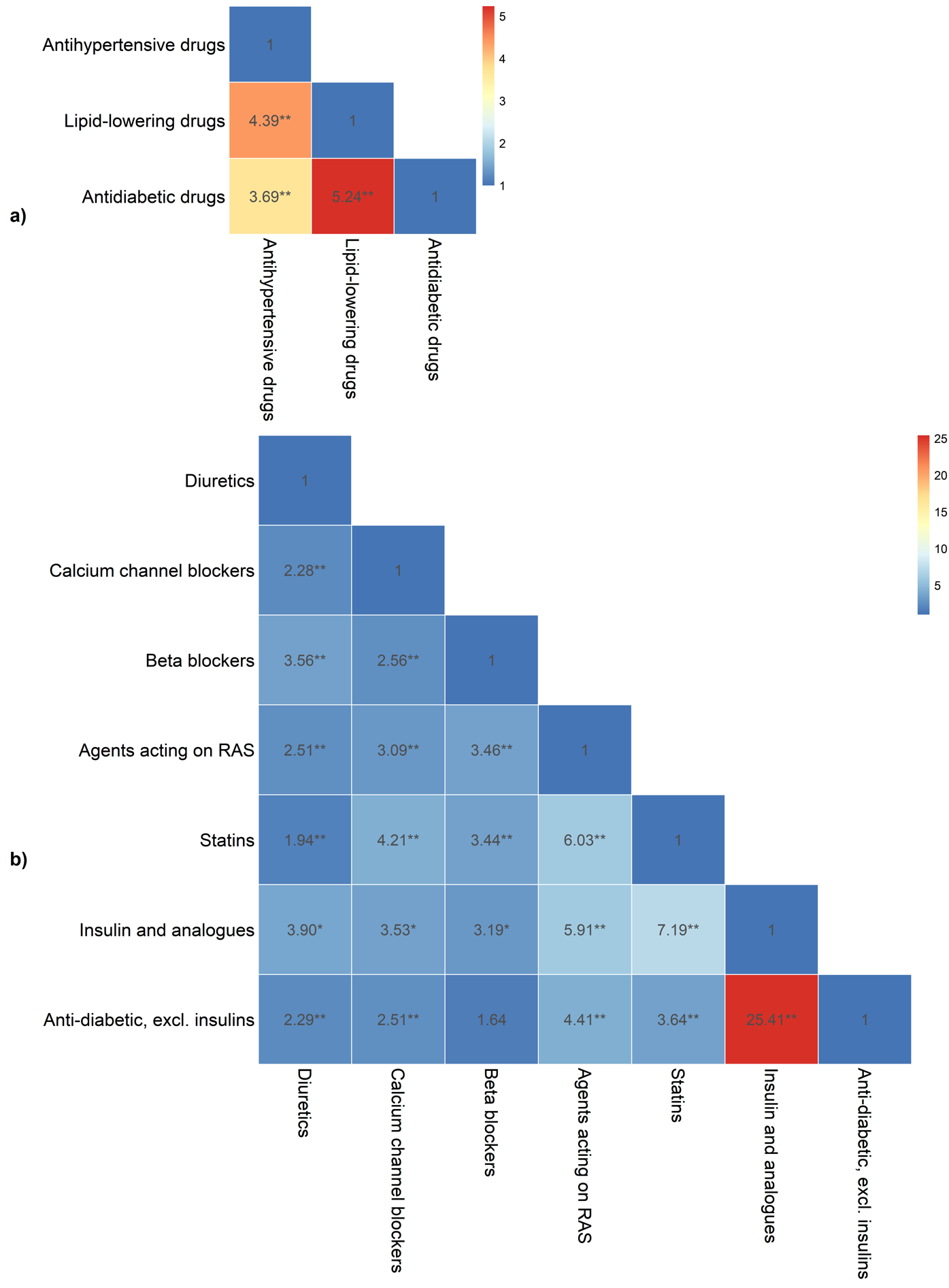

Supplement: Supplementary file 1 — (DOCX 15380 kb) [file 11357_2023_784_MOESM1_ESM.docx]
